# A Preliminary Analysis of Wind Retrieval, Based on GF-3 Wave Mode Data

**DOI:** 10.3390/s18051604

**Published:** 2018-05-17

**Authors:** Lei Wang, Bing Han, Xinzhe Yuan, Bin Lei, Chibiao Ding, Yulin Yao, Qi Chen

**Affiliations:** 1 Key Laboratory of Technology in Geo-Spatial Information Processing and Application Systems, Institute of Electronics, Chinese Academy of Sciences, Beijing 100190, China; wanglei162@mails.ucas.edu.cn (L.W.); leibin@mail.ie.ac.cn (B.L.); cbding@mail.ie.ac.cn (C.D.); 2 Institute of Electronics, Chinese Academy of Sciences, Beijing 100190, China; 3 School of Electronic, Electrical and Communication Engineering, University of Chinese Academy of Sciences, Beijing 100049, China; 4 National Satellite Ocean Application Service, State Oceanic Administration, Beijing 100081, China; harley_yuan@mail.nsoas.org.cn; 5 National Key Laboratory of Microwave Imaging Technology, Institute of Electronics, Chinese Academy of Sciences, Beijing 100190, China; 6 China Centre for Resource Satellite Data and Application, Beijing 100094, China; yaoyulin886@163.com (Y.Y.); chenq_cn@163.com (Q.C.)

**Keywords:** Gaofen-3, SAR, Wave Mode, calibration constants, cross-pol, noise floor, polarization ratio

## Abstract

This paper presents an analysis of measurements of the normalized radar cross-(NRCS) in Wave Mode for Chinese C-band Gaofen-3(GF-3) synthetic aperture radar (SAR). Based on 2779 images from GF-3 quad-polarization SAR in Wave Mode and collocated wind vectors from ERA-Interim, this experiment verifies the feasibility of using ocean surface wind fields and VV-polarized NRCS to perform normalized calibration. The method uses well-validated empirical C-band geophysical model function (CMOD4) to estimate the calibration constant for each beam. In addition, the relationship between cross-pol NRCS and wind vectors is discussed. The cross-pol NRCS increases linearly with wind speed and it is obviously modulated by the wind direction when the wind speed is greater than 8 m/s. Furthermore, the properties of the polarization ratio, denoted PR, are also investigated. The PR is dependent on incidence angle and azimuth angle. Two empirical models of the PR are fitted, one as a function of incidence angle only, the other with additional dependence on azimuth angle. Assessments show that the $\sigma_{\mathrm{VV}}^{0}$ retrieved from new PR models as well as $\sigma_{\mathrm{HH}}^{0}$ is in good agreement with $\sigma_{\mathrm{VV}}^{0}$ extracted from SAR images directly.

## 1. Introduction

With the continuous global depletion of petroleum energy, the development and utilization of clean wind energy have become a hot topic in recent decades. Offshore wind energy has been the focus of researchers due to the vast area of marine resources. Measurements of offshore wind information also contribute to oil spill monitoring, weather forecasting and understanding of air-sea interactions [1,2,3]. Spaceborne active microwave scatterometer such as QuickSCAT and ASCAT have provided mature wind products for National Oceanic and Atmospheric Administration (NOAA) [4,5]. However, the wind fields acquired by scatterometer cannot be applied to fine scale marine products due to the course spatial resolution (12.5 km~25 km) [6]. Because of features like imaging in all-weather conditions and high spatial resolution, synthetic aperture radar (SAR) has been widely used in military, economics, and science applications, and plays an important role in the retrieval of ocean surface wind fields, especially for C-band (~5.3 GHz) SAR [7].

Numerous studies have revealed that ocean surface normalized radar cross-section (NRCS) obtained from C-band SAR is mainly dominated by resonant Bragg backscattering at the centimeter scale wavelength [8,9,10]. This scale roughness is predominantly influenced by local wind and therefore ocean wind information may be extracted from SAR images [11]. In recent decades, several empirical geophysical model functions (GMFs), such as CMOD4 [12], CMOD_IFR2 [13], CMOD5 [14] and CMOD5.N [15] have been proposed to perform ocean surface wind retrieval. These GMFs relate the NRCS to the incidence angle, wind speed at a height of 10 m above sea level, and azimuth angle (radar look angle with respect to wind direction). Not only from scatterometers, such as QuickSCAT [16,17] and ASCAT [18], they can also accurately retrieve the wind speed from SAR images (within about 2 m/s), e.g., ENVISAT [19], RADARSAT-1/2 [20,21,22] and Sentinel-1A/B [23]. However, such GMFs are only suitable for VV-polarized NRCS, and no similar models exist to retrieve wind speed from images in HH-polarization. Therefore, it is necessary to convert HH-polarized NRCS to VV-polarization using polarization ratio (PR), denoted as $\mathrm{PR}=\sigma_{0}^{\mathrm{VV}}/\sigma_{0}^{\mathrm{HH}}$ [linear units], before retrieving wind speed [24,25,26,27,28]. In traditional research, it is generally believed that the PR is only relevant to incidence angle [24]. Recent studies in [27,28] show that the PR is also dependent on speed and azimuth. These results reveal that different satellites have their own optimal PR and GMF. Thus, the choice of suitable hybrid model is critical for Gaofen-3 satellite [29].

Recent decades, wind speed retrieval from cross polarized (cross-pol) NRCS has become a research focus due to the saturation of co-pol data at high wind speed. Some studies of cross-pol images have been conducted for RADARSAT-1/2 quad-polarization and dual-polarization [30,31,32,33,34,35,36,37]. Hwang and Zhang et al. [30,31] revealed the breaking contribution of cross-pol NRCS and emphasized the advantages of wind retrieval with cross-pol data at high wind speeds. Vachon and Zhang et al. [32,33] proposed a cross-pol wind retrieval model which is only relevant to wind speed and independent on incidence angle as well as wind direction, respectively. In [34,35,36], Hwang and Shen proposed that the VH NRCS of RADARSAT-2 dual-polarization mode is also relevant to incidence angle and the noise floor must be considered before wind retrieval. Moreover, Huang et al. [38] conducted an evaluation on cross-pol NRCS in Sentinel-1 IW mode and proposed a wind retrieval algorithm related to incidence angle and wind direction. The above studies show that the cross-pol NRCS has potential to retrieve high wind speeds, e.g., hurricanes and typhoons.

The accuracy of the retrieved wind vector is strongly affected by the absolute radiometric calibration accuracy of NRCS. Accurate wind speed can be obtained from refined NRCS. Therefore, it is possible to assess the accuracy of the calibration by using GMFs and known wind information. Horstmann et al. [39] propose a method for estimating ERS SAR calibration constants using C-band models and ocean surface wind fields. Stoffelen et al. [40] obtained an accurate calibration of a scatterometer over the ocean using CMOD4 and wind fields from European Centre for Medium-Range Weather Forecasts (ECMWF). This method achieves a calibration accuracy of 0.1 dB. Verspeek et al. [41] proposed an estimation correction table based on CMOD5.N to improve ASCAT wind retrieval. Zhu et al. [42] used Numerical Ocean Calibration (NOC) to calibrate HY-2 SCAT and the retrieved winds were in good agreement with winds from ECMWF.

The Gaofen-3 (GF-3) satellite, which was launched on 10 August 2016 by the China Academy of Space Technology (CAST), is the first C-band multi-polarization SAR in China with a highest resolution of 1 m. It has characteristics such as high resolution, large coverage, long-life operation and multiple imaging modes, including Wave Mode [43]. To date, some preliminary evaluations of ocean application have been carried out. Shao et al. [44] collected 244 Stander Stripmap (SS) and Quad-Polarization Stripmap (QPSI and QPSII) mode images to complete wind and wave retrieval firstly. In [29], Wang et al. validated the GF-3-derived winds against NDBC measurements using SS, QPSI, QPSII, FSI and NSC mode data. Ren et al. [45] conducted a comprehensive analysis of QPSI and QPSII mode data in each polarization. Several empirical algorithms for significant wave height in Wave Mode data and wind retrieval from cross-polarization in typhoons are also discussed in [46,47], which uses GF-3 images acquired in Global Observation (GLO) and Wide ScanSAR (WSC) mode.

The remainder of this paper is organized as follows: Section 2 describes the GF-3 Wave Mode SAR images and other validated data, including ECMWF ERA-Interim re-analysis wind fields and Amazon rainforest images. Methodologies for correcting calibration constants, fitting PR models and cross-pol wind speed retrieval formula are introduced in Section 3. Section 4 shows results of calibration, polarization conversion and wind speed retrieval accuracy. Finally, discussion and conclusion are presented in Section 5 and Section 6.

## 2. Description of Datasets

### 2.1. GF-3 SAR Wave Mode Images

An experiment in [48] shows that GF-3 images can meet the satellites’ polarimetric accuracy requirements, and the channel imbalance is 0.5 dB as well as a crosstalk accuracy of −35 dB. In this study, 6355 GF-3 Level-1A Wave Mode images are collected between 1 March 2017 and 31 December 2017 over the Pacific, Atlantic and Indian Ocean. The task of Wave Mode is to observe ocean surface waves over open ocean, and the size of Wave Mode image is about 5 km × 5 km every 50 km along the orbit. Incidence angle is the predominant difference between Wave Mode and other modes. Although the incidence angle of Wave Mode ranges from 20° to 50°, it only fixes in 28 beams with a narrow data acquisition window about 0.4°. This results in discrete incidence angle for images between different beams, e.g., incidence angle of beam 189 is about 21.5±0.2° and the incidence angle of beam 190 is about 23.7±0.2° etc. [43,46]. The parameter details of Wave Mode products are listed in Table 1 and the time distribution of data in each ocean is listed in Table 2.

The Level-1A products are single look complex (SLC) images. Let *I* represents real channel of images, *Q* as the imaginary channel. The equation of NRCS is as follows:

(1) $$\sigma^{0}=10*\log\left[{\left(\frac{I}{32767}\times \mathrm{Qualify}\right)}^{2}+{\left(\frac{Q}{32767}\times \mathrm{Qualify}\right)}^{2}\right]-K\_\mathrm{const}$$

where $\sigma^{0}$ is the NRCS in dB, Qualify is the QualifyValue in product description xml of GF-3, and K_const is the calibration constant.

Several studies indicate that wind speed can only be retrieved from pure ocean SAR images which are free of sea features not due to the local wind, e.g., ice and slicks [2,49]. To screen out the Wave Mode images which are not affected by features due to slicks or ocean phenomenon, the homogeneity check procedure proposed in [46] is used before the experimental study. Wang et al. [46] show that the Wave Mode normalized variance (cvar_vv) computed from VV-polarization images can be used as a standard for verifying image homogeneity. Here, we also choose the images which with 1.1<cvar_vv<1.6 for developing and validating wind retrieval algorithms. The parameter of homogeneity test is defined as:

(2) $$\mathrm{cvar}\_\mathrm{vv}=\mathrm{var}\left(\frac{I-\bar{I}}{\bar{I}}\right)$$

where $\bar{I}$ is the mean intensity of GF-3 Wave Mode image in VV-polarization. In addition, inappropriate receiver gain setting causes too high energy input to the ADC converter and may lead to saturation of output power [49]. It greatly affects the experimental results. Hence, this experiment only selects SAR images with 0% saturation coefficient which are provided in xml product description format.

**Figure 1:**
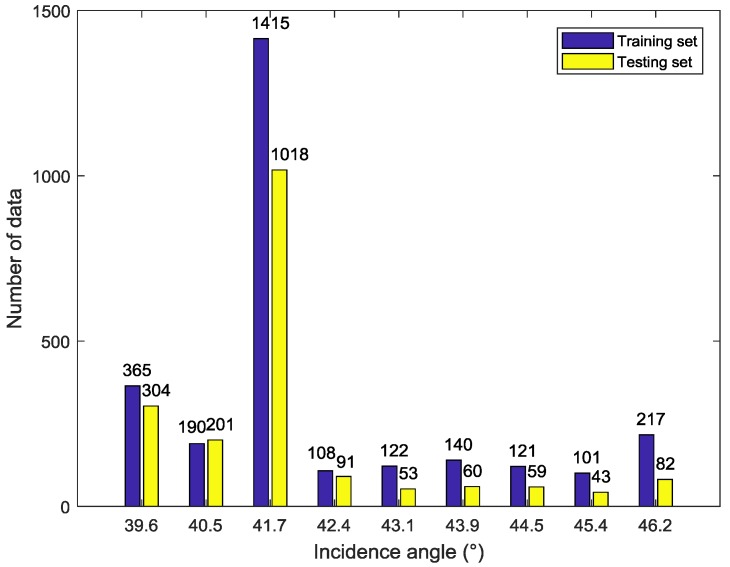
Incidence angle histogram of the data set.

**Figure 2:**
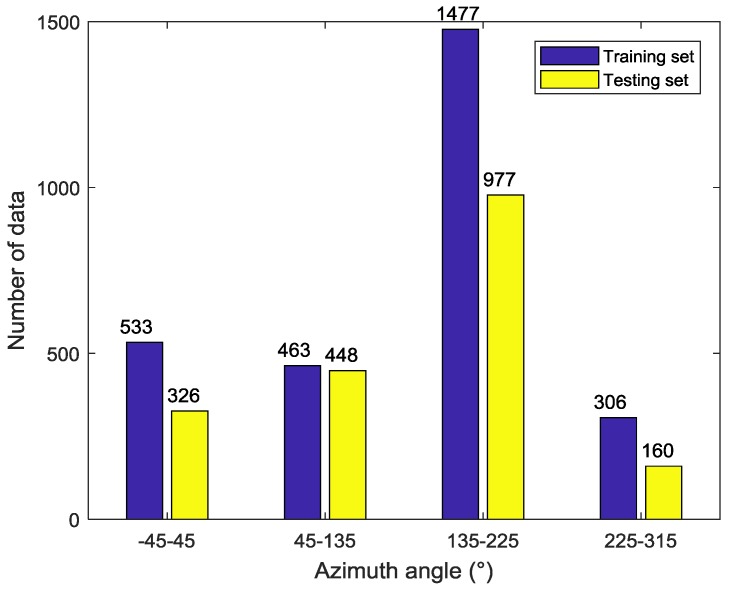
Azimuth angle histogram of the data set.

**Figure 3:**
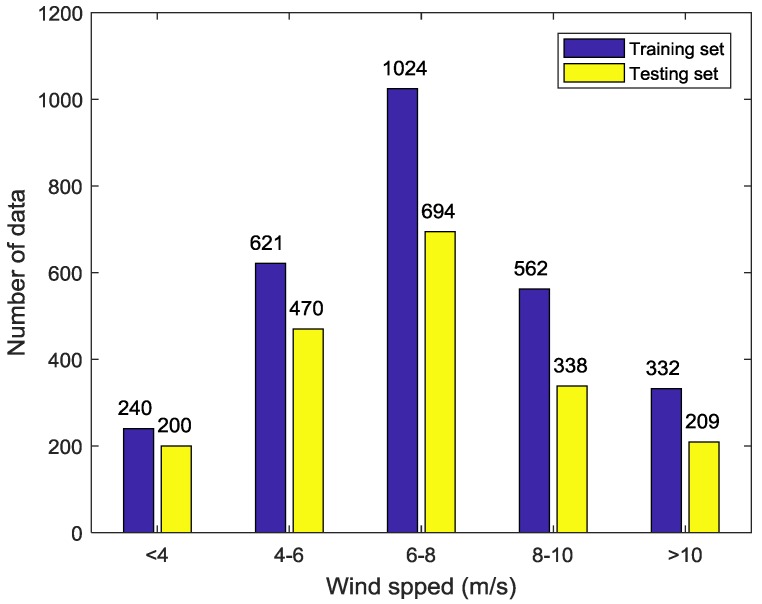
Wind speed histogram of the data set.

After the above two preprocessing processes, a total of 4690 GF-3 Wave Mode images are selected from 6355 images. The results show that the small incidence angle (in-angle < 36°) co-pol data is almost saturated, according to the xml product description. Therefore, the analyzed incidence angle of this paper is only from 39° to 47°. The detail information of data distribution is shown in the Figure 1, Figure 2 and Figure 3 below. To guarantee the validity of experiment, this experiment divides the whole data into training and testing set randomly first. Then, a small amount of data is adjusted artificially so that both sets can cover full range of incidence angles, azimuth angles and wind speeds. Finally, 2779 match-ups are chosen as training set and other 1911 samples as testing set.

### 2.2. Other Validation Sources

ERA-Interim is a global atmospheric re-analysis from 1979, continuously updated in real time, provided by ECMWF which is an independent intergovernmental organization supported by 34 states. The re-analysis wind field data is widely used in retrieval and comparison of wind vectors [12,13,14,15]. In this study, the spatial resolution of wind products downloaded on [50] is 0.125° × 0.125° (lat/lon), and the temporal resolution is 6 h (00:00, 06:00, 12:00, 18:00).

The Amazon rainforest has excellent temporal and spatial stability as a radar distributed target calibration source. And its maximum backscatter deviation is about 0.2 dB [51]. There have been numerous researches using the Amazon rainforest for radar radiometric calibration [52]. Here, this experiment uses beam 205 SAR images which have a large number of data and the Amazon rain forest Wave Mode SAR images of corresponding beam to validate the feasibility of ocean calibration.

## 3. Experiments and Analysis

For the 2779 training data and 1911 testing data, a 512 × 512 pixel boxcar is used in each center of Wave Mode images to average the NRCS in co-polarization (HH and VV), so that the NRCS spacing is about 5 km. As mentioned in Section 2, the wind fields’ spatial resolution is about 12.5 km × 12.5 km. To improve the match accuracy between wind fields and SAR images, we interpolate the wind fields time to 1 h using a cubic spline interpolation and use bilinear interpolation to interpolate four velocity components near the center point to the center. And the time difference between SAR image and wind vector is within 30 min.

### 3.1. Calibration Method Based on Ocean Wind

**Figure 4:**
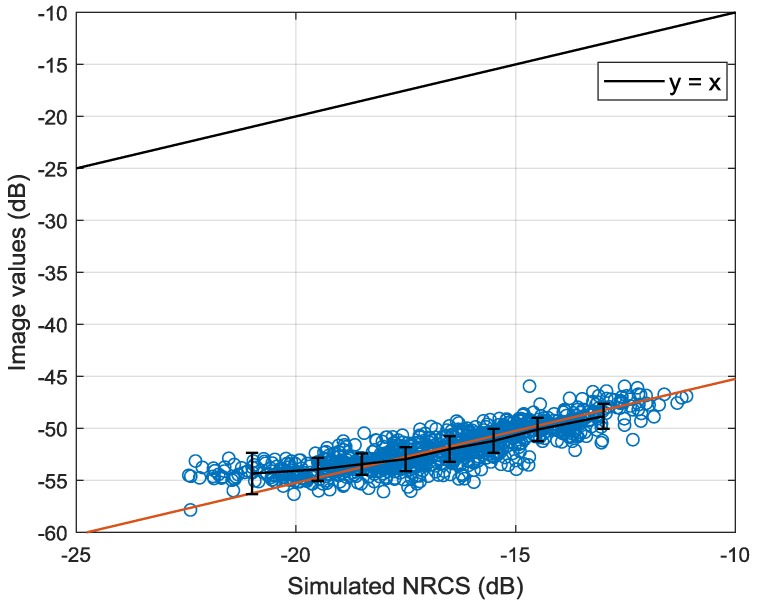
Relationship between simulated NRCS and values obtained directly from images.

As shown in Equation (1), the NRCS in dB is linear with the calibration constant. It provides a possibility for using the wind fields and GMFs to retrieve the calibration constant. In [53], it is found that CMOD4 has a better performance than CMOD5 under low to moderate wind speed. The wind speed range used in this paper is mainly focused on low to moderate wind speeds. Therefore, this experiment uses CMOD4 to obtain simulated VV-polarized NRCS. The difference between simulated NRCS and the value extracted directly from the corresponding image is the stimulated calibration constant. This method requires plentiful fitted data to ensure the accuracy of results and each beam has their own calibration constant. Therefore, the match-up data of 41.7° incidence angle (beam 205) in the training set is used to verify the calibration method. To guarantee the reliability of the calibration method, this experiment only selects data with wind speed higher than 4 m/s [39]. Moreover, the distribution of wind speed and direction in the experimental data set also affects the calculation of calibration constant. Hence, we first split the training set into wind speed bins of size 2 m/s and azimuth bins of size 90°. Then, let each speed bin has roughly the same amount of data and filter data in each speed bin so that the distribution of azimuth angle is uniform. Finally, 901 uniform match-ups are obtained to implement the calibration method. Figure 4 shows the relationship between simulated NRCS by CMOD4 and values obtained directly from VV polarized images. The error bars of bin 2 dB are also plotted.

The solid black line in Figure 4 is the bisector of the axis quadrant and the solid red line is the fitting curve of the training data with the same slope. As shown in the Figure, the difference between the simulated NRCS and image values is a constant. The best calibration constant is calculated using a minimum squared-error criterion. And the calculated calibration constant is 29.486. The calibration constant of beam 205 provided by China Centre for Resources Satellite Data and Application is 29.665. The difference between provided constant and calculated constant is within 0.2 dB. It shows the method has a good performance. The obtained calibration constants of each beam are listed in Appendix A.

### 3.2. Analysis of Wind Sensitivity for Cross-Pol NRCS

**Figure 5:**
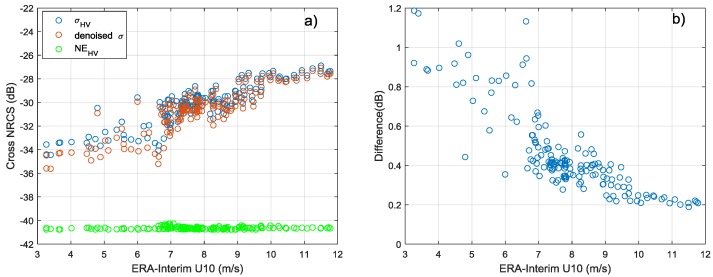
(**a**) the relationship between $\sigma_{\mathrm{HV}}^{0}$, denoised $\sigma_{\mathrm{HV}}^{0}$, noise floor and wind speed. (**b**) the difference between $\sigma_{\mathrm{HV}}^{0}$ and denoised $\sigma_{\mathrm{HV}}^{0}$.

The cross-pol backscattering signal-to-noise ratio (SNR) of ocean surface is much weaker than co-pol signal. Therefore, it is necessary to compare cross-pol NRCS with the system noise floor before wind retrieval [36]. The Chinese Academy of Sciences Institute of Electronics provides a ground system processing technology for GF-3 satellite and can obtain the noise gain coefficient of Wave Mode. Due to the limited number of products with system noise gain coefficient, only 138 sets of beam 205 match-ups with noise floor are collected. Figure 5a,b show the HV-polarized NRCS as a function of ERA-Interim re-analysis wind speed and the difference between $\sigma_{\mathrm{HV}}^{0}$ and $\sigma_{\mathrm{HV}}^{0}$ which is removed noise floor, respectively.

**Figure 6:**
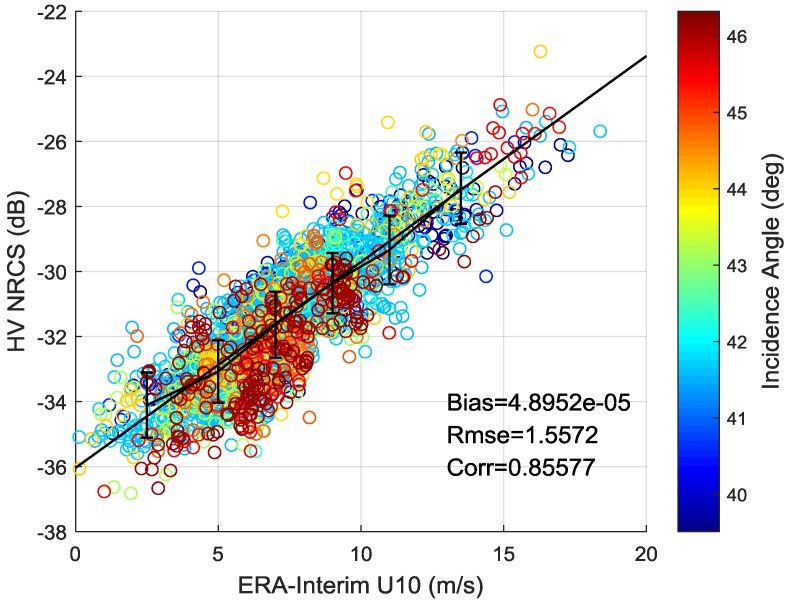
Relationship between NRCS and wind speed (different colors represent different incidence angle).

As illustrated in Figure 5a, the HV-polarized noise floor of beam 205 is about −40 dB. It is sufficiently low and the noise performance of GF-3 Wave Mode is better than RADARSAT-2 quad-polarization mode which has the noise floor of −36 dB [45]. Figure 5b shows that the noise floor has some effect on cross-pol signals at low wind speed (<10 m/s) and the difference between $\sigma_{\mathrm{HV}}^{0}$ and denoised $\sigma_{\mathrm{HV}}^{0}$ can be ignored at high wind speed. However, the number and distribution of noise floor is limited in this experiment. The noise floor of different beams may have some differences and $\sigma_{\mathrm{HV}}^{0}$ without noise removed also shows a clear linear relationship with wind speed. Therefore, this experiment temporarily ignores the effect of noise floor. The relationship between the NRCS $\sigma_{\mathrm{HV}}^{0}$ after calibration correction and wind speed is shown in Figure 6. Different colors represent different incidence angles. The errorbar of bin 2 m/s is also plotted. As shown in Figure 6, the $\sigma_{\mathrm{HV}}^{0}$ is independent on incidence angle and exists obvious linear relationship with wind speed. The black solid line is obtained using a non-linear least-squares method, and the formulation is:

(3) $$\sigma_{\mathrm{HV}}^{0}=0.6359\times U_{10}-36.1384$$

where $\sigma_{\mathrm{HV}}^{0}$ is the HV-polarized NRCS in dB and $U_{10}$ is the wind speed at 10 m.

The wind retrieval algorithm of cross-pol NRCS in this study is similar to the formula used in [32,33,45]. The wind speed retrieved using (3) has an RMSE of 1.56 m/s and a correlation coefficient of 0.86. It indicates the accuracy of cross-pol wind retrieval algorithm under low to moderate wind speed, and wind speed can be retrieved directly from cross-pol NRCS without inputting wind direction and incidence angle.

**Figure 7:**
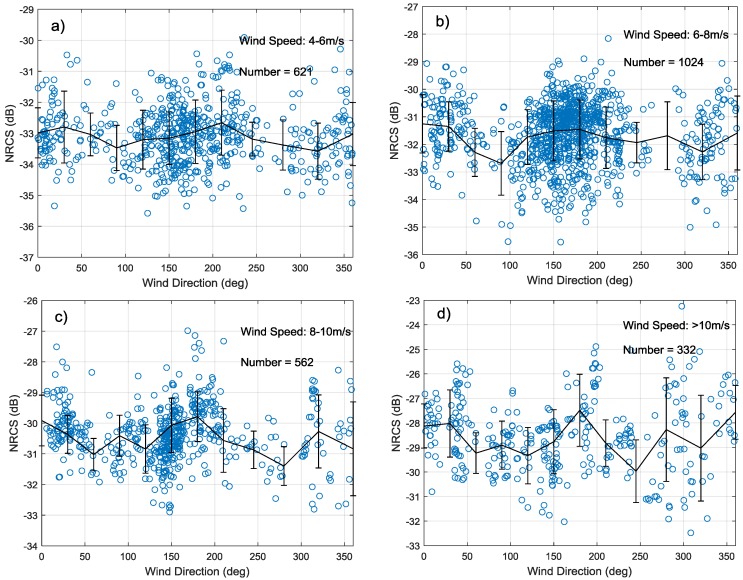
Relationship between cross-pol NRCS and azimuth angle at wind speed 4–6 m/s (**a**), 6–8 m/s (**b**), 8–10 m/s (**c**) and >10 m/s (**d**).

This paper also assesses the relationship between cross-pol NRCS and azimuth angle at different winds. The training set is divided into 4–6 m/s, 6–8 m/s, 8–10 m/s, >10 m/s four sets, respectively, and the variation trend of $\sigma_{\mathrm{HV}}^{0}$ with azimuth angle is shown in Figure 7a–d. Figure 7 also draw the mean value line at each bin 30° with error bars. When speed is higher than 8 m/s, the $\sigma_{\mathrm{HV}}^{0}$ is subject to obvious wind direction modulation and the maximum change is about 2.5 dB in different wind directions. This property is consistent with GF-3 QPSI and QPSII mode data in [45]. Therefore, the influence of wind direction should be considered when retrieving high wind speeds, e.g., hurricanes and typhoons.

### 3.3. Development of PR Models

**Figure 8:**
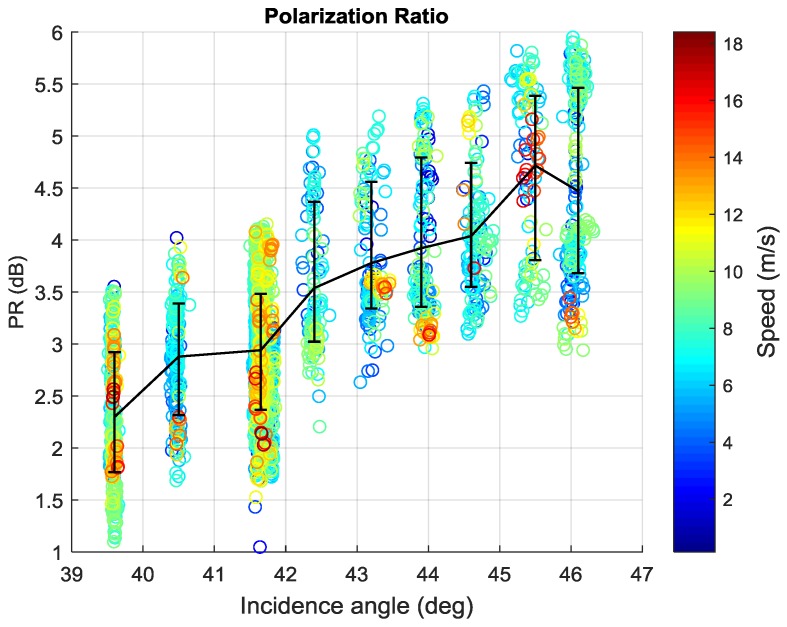
PR as a function of incidence angle (different colors represent different wind speed).

Figure 8 shows the relationship between PR and incidence angle as well as wind speed based on 2779 training data. Different colors represent the different wind speeds. The error bars of each incidence angle bin are also plotted. The PR rises rapidly with increasing incidence angle as in previous reports.

Here, A PR mode which is only related to the incidence angle is first fitted, defined as Model 1. The formula is:

(4) PR=Aexp(Bθ)+C

where PR is in linear unit, and A, B as well as C are coefficients fitted by a nonlinear least squares method. They are given in Table 3.

**Figure 9:**
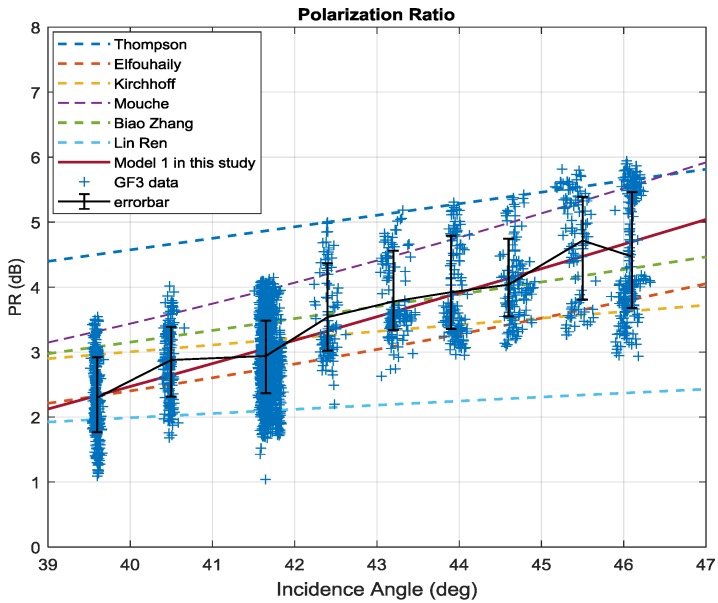
Comparison between Model 1 and other PR models.

Comparison with other PR models introduced in Section 1 is shown in Figure 9. PR models of Biao Zhang and Mouche [27,28] are also an exponential of the incidence angle like the one in present study, however different coefficients are found. The formula of other researchers [24,45] is expressed as:

(5) $$\mathrm{PR}={\left(1+2\tan^{2}\theta \right)}^{2}/{\left(1+{\alpha \tan}^{2}\theta \right)}^{2}$$

where α is an adjustable parameter. As illustrated in Figure 9, the Model 1 is closest to the mean of GF-3 Wave Mode.

**Figure 10:**
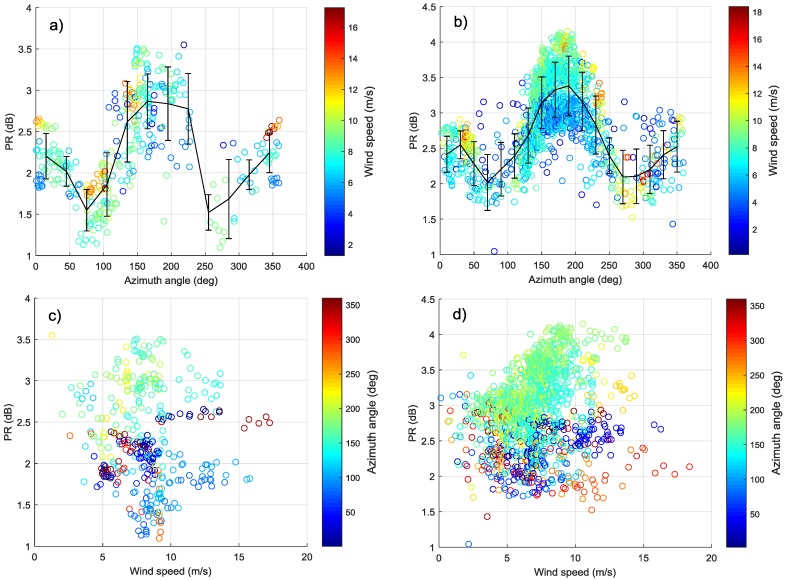
(**a**,**b**) represent the variation between PR and azimuth angle and different color shows different wind speed. (**c**,**d**) show the relationship between PR and wind speed. Different color represents different azimuth. (**a**,**c**) are at incidence angle 39.6°. (**b**,**d**) are at incidence angle 41.6°.

**Figure 11:**
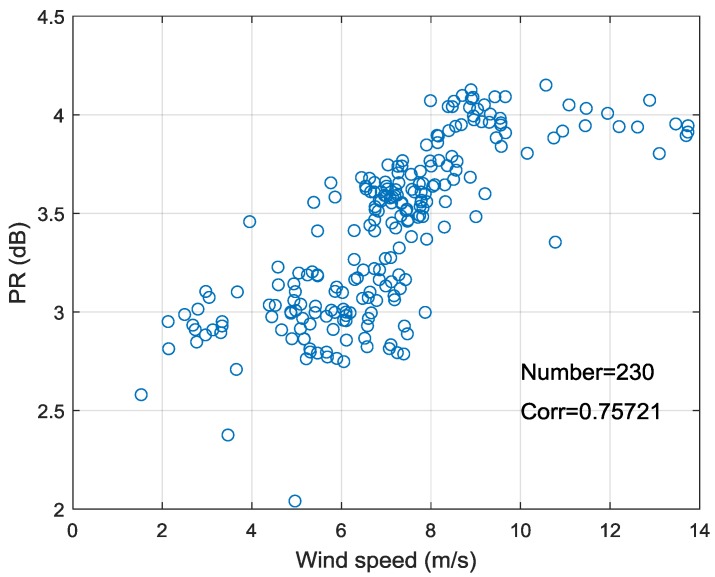
The relationship between PR and wind speed of beam 205 data in downwind.

To give a more comprehensive PR analysis for GF-3 Wave Mode data, the relationship between PR and azimuth angle is also studied. It shows a similar characteristic described in [28]. The variation of PR with azimuth angle and wind speed is shown in Figure 10a–d at the incidence angle of 39.6° and 41.7° (beam 202 and 205). The error bars of bin 30° and 20° are plotted in Figure 10a,b respectively. Figure 10a,b show that there is an approximate cosine relationship between PR and azimuth angle like the characteristic between NRCS and azimuth angle. The maximum of PR is observed in downwind direction (ϕ=180°), a secondary maximum is appeared in upwind direction (ϕ=0°) and the minimum values are in crosswind (ϕ=90°). This is slightly different from NRCS, which appears maximum in upwind and secondary maximum value in downwind. In addition, Figure 10c,d show the PR tends to increase with the increase of wind speed (<10 m/s) in the downwind, while it is independent with wind speed in other wind direction. To more clearly analyze the variation of PR with wind speed in downwind, this experiment screens out beam 205 data with azimuth angle of 180±10°, and the relationship between the PR and wind speed is plotted in Figure 11. The correlation coefficient (0.7572) between PR and wind speed illustrates that the PR is positively related to the wind speed in downwind.

However, it cannot be concluded that the PR increases linearly with wind speed due to insufficient high wind speed data in downwind. Hence, this experiment temporarily ignores the influence of wind speed and fits training set using nonlinear least squares, deriving Model 2 for PR with additional dependence on azimuth angle. The Model 2 is assumed to follow the expression:

(6) $$\mathrm{PR}\left(\theta ,\phi \right)=C_{0}\left(\theta \right)+C_{1}\left(\theta \right)\cos\phi +C_{2}\left(\theta \right)\cos2\phi$$

where ϕ is azimuth angle. In each azimuth angle, the relationship between PR and incidence angle is also defined as exponential function:

(7) $${\mathrm{PR}}_{\phi }\left(\theta \right)=A_{\phi }\exp\left(B_{\phi }\theta \right)+C_{\phi }$$

The coefficients $C_{i}$(i = 0, 1, 2) can be calculated by the method of undetermined coefficients, and the formulas are as follows:

(8a) $$C_{0}\left(\theta \right)=\left(\mathrm{PR}\left(\theta ,0\right)+\mathrm{PR}\left(\theta ,\pi \right)+2\mathrm{PR}\left(\theta ,\pi /2\right)\right)/4$$

(8b) $$C_{1}\left(\theta \right)=\left(\mathrm{PR}\left(\theta ,0\right)-\mathrm{PR}\left(\theta ,\pi \right)\right)/2$$

(8c) $$C_{2}\left(\theta \right)=\left(\mathrm{PR}\left(\theta ,0\right)+\mathrm{PR}\left(\theta ,\pi \right)-2\mathrm{PR}\left(\theta ,\pi /2\right)\right)/4$$

First, the coefficients ($A_{\phi }$, $B_{\phi }$, $C_{\phi }$) of three main directions (upwind, downwind, crosswind) are fitted using a nonlinear least squares method. Then using them to obtain coefficients $C_{i}$. Table 4 shows the fitting results.

## 4. Validation and Results

### 4.1. Results of Ocean Calibration

**Figure 12:**
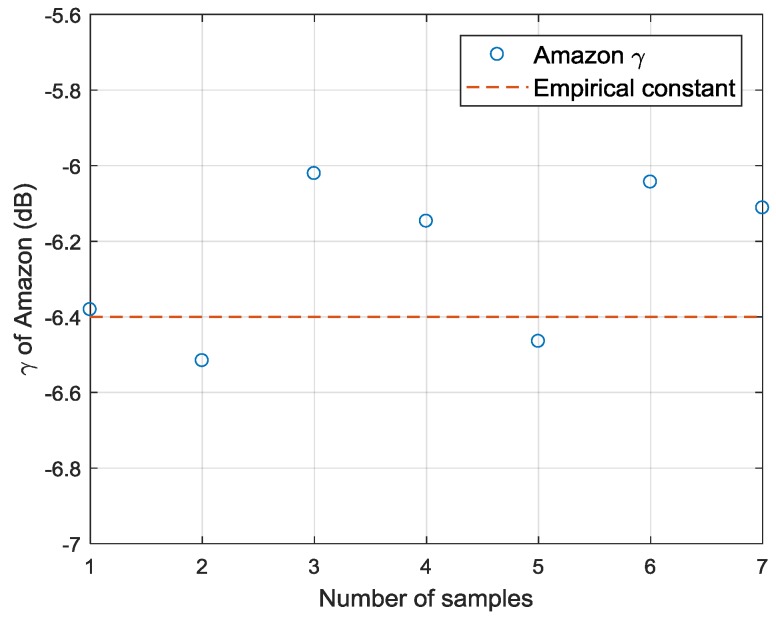
Distribution of Amazon rainforest γ.

The calibration constant provided by China Centre for Resources Satellite Data and Application is derived from system bandwidth and antenna pattern and has not been verified by field calibration. Therefore, 7 GF-3 Wave Mode SAR images of the Amazon rainforest area in beam 205 are collected to verify the calibration constant obtained in Section 3.2. The distribution of Amazon rainforest γ is shown in Figure 12.

As described in [52], the γ of Amazon rainforest can be considered as a constant value due to the stability of this area and it is independent on incidence angle. The γ can be characterized as:

(9) $$\gamma =\sigma^{0}/\cos\theta =\beta^{0}\tan\theta$$

where γ describes the reflectivity of distributed scatterers per unit area of the incident wave front, $\sigma^{0}$ describes the reflectivity per unit area of horizontal surface and $\beta^{0}$ describes the radar reflectivity per unit pixel area [54]. It is generally accepted that the γ of Amazon rainforest is around −6.4 dB. And the distribution of γ from RADARSAT-1 is −6.47±0.71 dB according to [52]. Figure 12 illustrates the γ of GF-3 Wave Mode data in beam 205 is around −6.4±0.4 dB. Therefore, it can be demonstrated that the calibration constant calculated using the ocean calibration is accurate. And if enough data can be acquired, the calibration constant can be obtained continuously using the ocean surface wind fields. It provides the possibility for normalized calibration.

**Figure 13:**
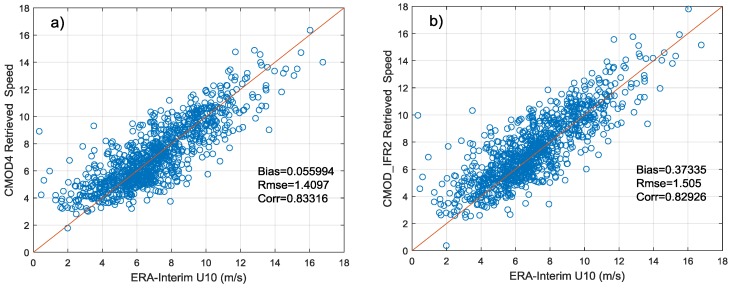

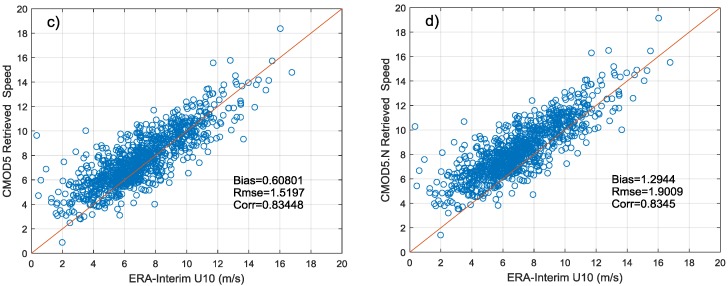
Comparison of ERA-Interim U10 with SAR-derived wind speeds which use CMOD4 (**a**), CMOD_IFR2 (**b**), CMOD5 (**c**) and CMOD5.N (**d**).

Based on the obtained calibration constant, this experiment uses GMFs to perform wind speed retrieval on beam 205 data of testing set. Figure 13a–d show the comparison between ERA-Interim wind speeds and retrieved wind speeds using CMOD4, CMOD_IFR2, CMOD5, CMOD5.N, respectively.

As demonstrated in Figure 13, the estimated calibration constant can be well applied to SAR image wind speed retrieval and the RMSE of retrieved speed is less than 2 m/s. However, large inaccuracies may occur in wind retrieval using GMFs when the wind speed is lower than 2 m/s. The accuracy of retrieved speed using CMOD4 is higher than others at low to moderate wind speeds and its RMSE is 1.41 m/s. The advantage of CMOD5 cannot be verified due to the lack of data when the wind speed comes too high.

### 4.2. Validation of Wind Retrieval for Cross-Polarization

The testing set is used to evaluate the performance of cross-polarization wind retrieval formula in this paper compared with algorithms in [32,33,45]. The RMSE, bias and R-squares between ERA-Interim U10 and retrieved speed are listed in Table 5.

As shown in Table 5, the algorithm fitted in this paper has the optimal inversion accuracy with RMSE 1.499 m/s. The formula proposed by Zhang has the smallest bias with −0.0106 m/s. The retrieval result is slightly poor when the formula fitted by QPSI and QPSII data is applied to the Wave Mode data, which has RMSE with 2.04 m/s and bias with −1.16 m/s. This experiment uses the calibration constants after correction in cross-pol wind retrieval. Therefore, the calibration constants used here may have some differences with Ren. And the noise floor of QPSI, QPSII and Wave Mode is also slightly different. These may cause that the cross-pol retrieval method of Ren shows a different accuracy compared to the formula fitted in this paper.

### 4.3. Validation of PR Models Using Testing Set

To evaluate the performance of two fitted PR models, we test the models in testing set and compare two models with different models in [27,45]. PR model proposed by Zhang in [27] is a function of incidence angle as well as wind speed and model fitted by Ren in [45] is dedicated to GF-3 QPSI and QPSII mode data.

**Figure 14:**
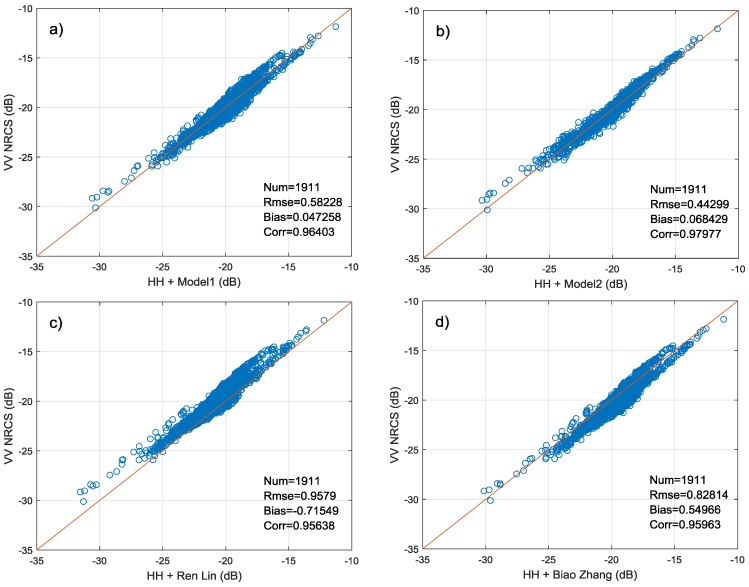
Comparison of four different PR models based on testing set where (**a**) represents PR Model1, (**b**) represents PR Model2, (**c**) represents model proposed by Ren and (**d**) represents model proposed by Zhang.

Figure 14a–d illustrate the comparison of four PR models. The abscissa of figure is retrieved NRCS by PR model and the ordinate is NRCS from SAR image in VV polarization. Figure 14 also show the root-mean-square error (RMSE), bias and correlation coefficient for each model. It is shown that two models fitted in this study are in better agreement with Wave Mode data. The bias of Model 1 and Model 2 is much lower than two other models. And Model 2 which considers the influence of wind direction has a smaller RMSE (0.443 dB) and larger correlation coefficient (0.98). In addition, Figure 14c shows the PR model proposed for GF-3 QPSI and QPSII mode cannot be used well in Wave Mode data. The retrieved NRCS is generally lower than observation. There may be two reasons for this result. First, the imaging bandwidth and system noise floor of two operating modes are different. These may affect the observation of NRCS. In addition, the PR model in Figure 14c is mainly fitted by data with incidence angles between 35°–38°, while PR models in this study are mainly applicable to data with incidence angles greater than 39°, due to the lack of data of small incidence angle.

## 5. Discussion

In this paper, we conduct a preliminary analysis of SAR images in Wave Mode for GF-3 satellite. 2779 GF-3 Wave Mode NRCS and wind vectors for the corresponding location are collected as training set and additional 1911 match-ups are as testing set. To reduce the effect of speckle noise and improve the matching precision of the data set, the NRCS is first sampled from 10 m pixel spacing to 5 km firstly, then the wind field interval is interpolated to 1 h and the wind vectors of the center of each SAR image is calculated using bilinear interpolation.

A simple method for absolute radiometric calibration using ocean surface wind fields and CMOD4 is introduced and tested. Due to the linear relationship between NRCS and calibration constant, an estimator of calibration constant can be obtained by calculating the difference between the simulated NRCS and image value. Since the calibration constant given by China Centre for Resources Satellite Data and Application is only calculated by combing system bandwidth and antenna pattern, this experiment also verifies the constant using Amazon rainforest data. The result shows the obtained Amazon rainforest γ using estimated calibration constant is in good agreement with empirical γ. This normalized calibration method provides a more convenient and affordable way for future absolute radiometric calibration. It saves the expensive cost of calibration using corner reflector and can obtain an accurate calibration constant continuously.

The relationship between cross-pol images of Wave Mode and system noise floor, wind vectors and satellite geometry parameters is also investigated. The experiment indicates that the system noise floor of cross polarization is about −40 dB. It is low enough and stable. There is a clear linear relationship between cross-pol NRCS and wind speeds in the case of ignoring noise floor effects, and the cross-pol NRCS is independent on incidence angle. As the wind speed increase, the cross-pol NRCS is more affected by azimuth angle. Therefore, it is necessary to consider the azimuth angle when retrieving high wind speed in the future.

The PR of Wave Mode is not only dependent on incidence angle but also modulated by the azimuth angle. Its first maximum corresponds to downwind direction, the second maximum in the upwind, and two minima appear in the crosswind direction. Moreover, when speed is lower than 10 m/s, the PR presents a linear increase trend with wind speed in the downwind while it is independent on wind speed in other wind directions. Therefore, we fit two PR models which are suitable for large incidence angle using training set. The first is only dependent on incidence angle (Model 1) and the other one adds additional azimuth angle variable (Model 2). The results of two models on the testing set show that the PR models fitted in this paper are superior to models given in previous studies [24,25,26,27,28,45]. The Model 2 has higher polarization conversion accuracy than Model 1, with RMSE 0.443 dB and correlation coefficient 0.98.

## 6. Conclusions

To date, the GF-3 satellite has only been in operation for less than two years and is still in the preliminary application stage. Since SAR images before February 2017 lack saturation coefficients and it cannot be confirmed whether the data is saturated or not, the images used in this paper are all collected after March. Furthermore, as shown in the Table 2, the temporal and spatial distribution of SAR images are not uniform. Most data in the experiment is from the east Pacific Ocean near North America in September, October and November. The three-month data is mainly concentrated in beam 205 (41.7°). Therefore, the beam 205 is the main part of the data. The collected SAR images whose incidence angles are lower than 39° (lower than beam 200) are concentrated in March and April. They are all saturated and cannot be used. The reason for this may be inappropriate receiver gain setting at initial period of satellite operation. These lead to the non-uniformity distribution of incidence angle. In addition, we use the calibration constants after correction in cross-pol wind retrieval. And the noise floor of QPSI, QPII and Wave Mode is also slightly different. These may cause that cross-pol retrieval method of Ren shows a quite different accuracy compared to mine.

In the future work, we will collect more Wave Mode images which cover a wide range of incidence angles and wind speeds in high wind conditions. More system noise gain coefficient files will also be obtained to analyze the influence of noise floor on cross-pol wind speed retrieval. Moreover, we will further research the reasons for different polarization ratios under different operating modes to find a uniform PR model for GF-3 satellite.

## Figures and Tables

**Table 1:** Parameters for GF-3 Wave Mode.

Imaging Mode	Incidence Angle (°)	Polarization	Resolution (m)	Swath (km)
WAV	20–50	HH + VV + HV + VH	10	5

**Table 2:** Time Distribution of SAR Images in Each Ocean.

Oceans	Pacific	Atlantic	Indian
**Distribution**	March, April, September, October, November, December	April, May, June	March, April

**Table 3:** Coefficients of Model 1.

Cofficient	Fitted Values
A	0.02985
B	0.09727
C	0.305

**Table 4:** Coefficients of Model 2.

Coefficients	Fitted Values
$A_{0}$	0.1715
$B_{0}$	0.06242
$C_{0}$	−0.4342
$A_{\pi /2}$	0.9331
$B_{\pi /2}$	0.03606
$C_{\pi /2}$	−2.44
$A_{\pi }$	0.000393
$B_{\pi }$	0.1912
$C_{\pi }$	1.119

**Table 5:** Comparison of Wind Speed Retrieval Algorithm.

	RMSE (m/s)	Bias (m/s)	R-Square
**Mine**	1.4990	−0.1605	0.6310
**Vachon**	1.6043	0.2191	0.5773
**Zhang**	1.6227	−0.0106	0.5675
**Ren**	2.0371	−1.1586	0.3184

## Appendix A

**Table A1:** Calibration Constants of Oher Beams.

Beams	Constants
202	28.966
203	28.738
206	28.366
207	27.836
208	27.105
209	27.538
210	27.854
211	27.809

**Table A2:** Index of Abbreviations and Notations.

Abbreviations	Full Name
GF-3	Gaofen-3
NOAA	National Oceanic and Atmospheric Administration
SAR	Synthetic Aperture Radar
ECMWF	European Centre for Medium-Range Weather Forecasts
GMFs	empirical Geophysical Model Functions
NRCS	normalized radar cross-section
PR	polarization ratio
NOC	Numerical Ocean Calibration
CAST	China Academy of Space Technology
SLC	single look complex
SNR	signal-to-noise ratio
